# Molecularly Imprinted Drug Carrier for Lamotrigine—Design, Synthesis, and Characterization of Physicochemical Parameters

**DOI:** 10.3390/ijms25094605

**Published:** 2024-04-23

**Authors:** Monika Sobiech, Sandile M. Khamanga, Karol Synoradzki, Tamara J. Bednarchuk, Katarzyna Sikora, Piotr Luliński, Joanna Giebułtowicz

**Affiliations:** 1 Department of Organic and Physical Chemistry, Faculty of Pharmacy, Medical University of Warsaw, Banacha 1, 02-097 Warsaw, Poland; monika.sobiech@wum.edu.pl (M.S.); katarzynaa.sikoraa@gmail.com (K.S.); 2 Faculty of Pharmacy, Rhodes University, Makhanda 6140, South Africa; s.khamanga@ru.ac.za; 3 Institute of Molecular Physics, Polish Academy of Sciences, Smoluchowskiego 17, 60-179 Poznań, Poland; karol.synoradzki@ifmpan.poznan.pl; 4 Institute of Low Temperature and Structure Research, Polish Academy of Sciences, Okólna 2, 50-422 Wrocław, Poland; t.bednarchuk@intibs.pl; 5 Department of Drug Chemistry, Pharmaceutical and Biomedical Analysis, Faculty of Pharmacy, Medical University of Warsaw, Banacha 1, 02-097 Warsaw, Poland; joanna.giebultowicz@wum.edu.pl

**Keywords:** molecularly imprinted polymer, magnetic particle, drug carrier, lamotrigine, drug delivery

## Abstract

This study presents the initial attempt at introducing a magnetic molecularly imprinted polymer (MIP) designed specifically for lamotrigine with the purpose of functioning as a drug carrier. First, the composition of the magnetic polymer underwent optimization based on bulk polymer adsorption studies and theoretical analyses. The magnetic MIP was synthesized from itaconic acid and ethylene glycol dimethacrylate exhibiting a drug loading capacity of 3.4 ± 0.9 μg g^−1^. Structural characterization was performed using powder X-ray diffraction analysis, vibrating sample magnetometry, and Fourier transform infrared spectroscopy. The resulting MIP demonstrated controlled drug released characteristics without a burst effect in the phospahe buffer saline at pH 5 and 8. These findings hold promise for the potential nasal administration of lamotrigine in future applications.

## 1. Introduction

Lamotrigine (6-(2,3-dichlorophenyl)-1,2,4-triazine-3,5-diamine) is an antiepileptic drug effective against stiffening and rhythmical convulsions, as well as in the treatment of psychiatric disorders and mood stabilization. The mechanism of lamotrigine action is related to the inactivation of voltage-dependent sodium channels, preventing the release of excitatory neurotransmitters associated with epilepsy [1,2]. Initially, the drug was claimed to be ideal for treating epilepsy in pregnant women, but pharmaco-resistance, related to the activation of brain glycoproteins by lamotrigine, resulted in limited permeation through the blood–brain barrier, leading to a decrease in therapeutic effects. Moreover, side effects such as severe cutaneous adverse reactions induced by lamotrigine (anticonvulsant hypersensitivity syndrome or toxic epidermal necrolysis) and induced tic disorders limited its pharmacotherapy [3,4]. These effects could be related to an overdose of lamotrigine and non-linear elimination of the drug [5]. Lamotrigine is a lipophilic weak base with good oral bioavailability, a peak plasma concentration after 1–3 h, a volume of distribution of 1.0–1.3 L kg^−1^, a protein binding at the level of 55%, and therapeutic plasma concentrations of 2.5–15 mg L^−1^. The elimination process occurs by hepatic glucuronidation with urinary excretion, requiring patients with hepatic failure to reduce the drug dose by at least 50% [6,7]. Oral administration is the main application route, and the dosage of lamotrigine is set to 25 mg per day. Nevertheless, its low aqueous solubility at a level of 0.17 g L^−1^ may result in the delayed onset of action due to sub-therapeutic plasma drug levels, and may also lead to therapeutic failure [8]. For that purpose, new forms of lamotrigine delivery such as nanoliposomes [9], polymeric nanoparticles [10], multiple-unit beads [11], or co-crystals [12], as well as alternative routes of its administration, such as intrathecal [13], transdermal [14], or nasal [15], have recently become a hot topic of scientific investigations. Recently, the latter administration route has gained particular attention due to the direct delivery of lamotrigine to the brain [15], but it suffers from fast clearance from the nasal cavity due to mucociliary action, susceptibility to enzymes present in the nasal mucosa, low absorption of polar drugs and macromolecules, lower bioavailability in case of cold or allergies interferes, and irritation to the mucosa [16]. To overcome existing problems, novel drug vehicles with modified releasing properties and high specificity should be explored. Among advanced approaches are cyclodextrin-based nanosponge delivery systems [17], silk sutures coated with wax and halloysite nanotubes [18], hybrid hydrogels composed of halloysite nanotubes and alginate [19], or magnetic hydroxyapatite nanoparticles [20]. Here, molecularly imprinted polymers (MIPs) could be considered as an alternative due to advantages such as high selectivity, high durability, and facile capability for functionalization of other materials, such as magnetic nanoparticles. These properties determined the prevalent application of MIPs for separation and detection purposes [21]. A few of them were dedicated to the determination of lamotrigine [22,23,24,25,26]. However, to the best of our knowledge, the evaluation of molecularly imprinted drug carriers for lamotrigine has not been reported. It should be emphasized that the potential of MIPs as drug vehicles has been recently investigated, providing novel ideas and interesting approaches such as magnetic molecularly imprinted polymer of polydopamine/graphene oxide as a drug carrier for rivastigmine [27], (2-hydroxyethyl methacrylate)/chitosan nanocarrier for cefixime [28], or magnetite surface-grafted carboxymethyl/chitosan molecularly imprinted polymer for the delivery of salidroside [29]. Recent reviews have summarized current achievements in the field [30,31]. However, it must be underlined that the most rapidly expanding area for the application of molecularly imprinted polymer drug carriers is related to cancer therapy and diagnosis [32]. Here, the molecularly imprinted polymer for 4-borono-L-phenylalanine in boron neutron capture therapy [33], the magnetic molecularly imprinted carrier for the targeted delivery of the anticancer drug docetaxel [34], the pH-responsive magnetic molecularly imprinted polymer [35], or zeolitic imidazolate framework-8 molecularly imprinted polymer [36] for prostate cancer therapy could serve as very interesting examples.

In this study, a molecularly imprinted drug carrier for lamotrigine was designed, synthesized, and characterized. Theoretical analysis was utilized to preselect the most effective polymeric system prior to the synthesis of bulk MIPs. The theory also enabled us to elucidate the interactions between the drug and the monomer residues in the polymer network. The preselected polymeric system was employed to synthesize a magnetic core-shell material with a MIP external layer. Characterization confirmed the composition, structure, and morphology of the material. The main objective of the research was to verify the capability of the magnetic MIP carrier for the release of lamotrigine. The results obtained in this study could be of great interest for the development of a lamotrigine drug delivery system for administration via the nasal route.

## 2. Results and Discussion

### 2.1. Composition of the Polymer

#### 2.1.1. Analysis of Sorption Properties of Polymers

In the first step of preparing the molecularly imprinted drug carrier for lamotrigine, the composition of the MIP was optimized. It is well known that the binding capacity is determined by the presence of monomer residues in the polymer matrix. Considering the application of MIP for drug delivery, both satisfactory binding capacity and selectivity should be considered. For this purpose, five functional monomers with different physicochemical properties were investigated in the formation of the polymer matrix, namely 2-hydroxyethyl methacrylate (**1**), 4-vinylpyridine (**2**), methacrylic acid (**3**), 4-vinylbenzoic acid (**4**), and itaconic acid (**5**). Bulk MIPs were obtained using ethylene glycol dimethacrylate (EGDMA) as the cross-linking agent. Additionally, the polymers were synthesized in the presence of 2,4-diamine-1,3,5-triazine as the template molecule. The template molecule possesses similar characteristics to the lamotrigine molecule (though not identical) with two amine groups substituted into the heteroaromatic ring, allowing the formation of stable complexes with selected functional monomers prior to their incorporation into the MIP. It is worth emphasizing that the use of a structural analog of the drug, namely lamotrigine, is strongly recommended to avoid leaching of pharmacologically active components from the MIP when using them as drug carriers.

Subsequently, the binding capacities (*B*, µmol g^−1^) of the resulting bulk polymers MIP**1**–MIP**5** and NIP**1**–NIP**5** were determined, and the selectivity was calculated according to Equations (1)–(3). The results are presented in Table 1.

As observed, the binding capacities of the resulting polymers varied significantly. Among the MIPs, the lowest binding capacity was determined for a polymer prepared from 2-hydroxyethyl methacrylate. This could be attributed to the weak interactions between lamotrigine and monomer residues during the adsorption process. It is worth noting that only a slightly higher binding capacity was observed for the polymer prepared from 4-vinylpyridine. Significantly higher binding capacities were observed for MIPs prepared from acidic functional monomers, with the highest value noted for MIP**5**, which possessed two carboxylic residues from the itaconic acid monomer. The binding capacity of MIP**5** was four times higher than that observed for MIP**1**. This could be explained by the presence of strong interactions between lamotrigine and the two carboxylic residues in MIP**5**. Moreover, it should be emphasized that the strong interactions between the template and monomer stabilized the prepolymerization complex, affecting the surface modification of MIP**5** and resulting in the formation of well-defined cavities in MIP**5** after the template removal process. Additionally, it should be noted that MIP**5** exhibited the highest selectivity (IF = 6.62).

#### 2.1.2. Theoretical Evaluation of Interactions in the MIPs Cavity

Optimization of the MIP synthetic procedure is a crucial step in obtaining a material with the most favorable properties for selective sorption of the chosen compound. Computational methods can aid in this optimization process by reducing the number of experimental tests, which not only benefits the environment but also saves financial and time resources. During the theoretical analysis, we tested five monomers (**1**–**5**) and prepared five models of the polymeric matrix (MIP**1**–MIP**5**), which were analyzed for their affinity properties towards lamotrigine. To mimic the molar ratio used in the synthetic procedure, we employed two template molecules, eight monomer molecules, and forty cross-linker molecules in the binding site creation process. Additionally, by considering two template molecules, we aimed to analyze the probability of template–template interactions and their impact on the creation of binding cavities in the polymer and on the sorption properties.

In the models of prepolymerization complexes, we observed that in MIP**1**, MIP**2**, and MIP**5**, the template molecules interacted with each other, resulting in non-classical hydrogen bond formation in MIP**1**, π–π stacking interaction in MIP**2** and MIP**5**, and π–lone pair interaction in MIP**5**. No interactions between the template molecules were present in the MIP**3** and MIP**4** systems.

**Figure 1:**
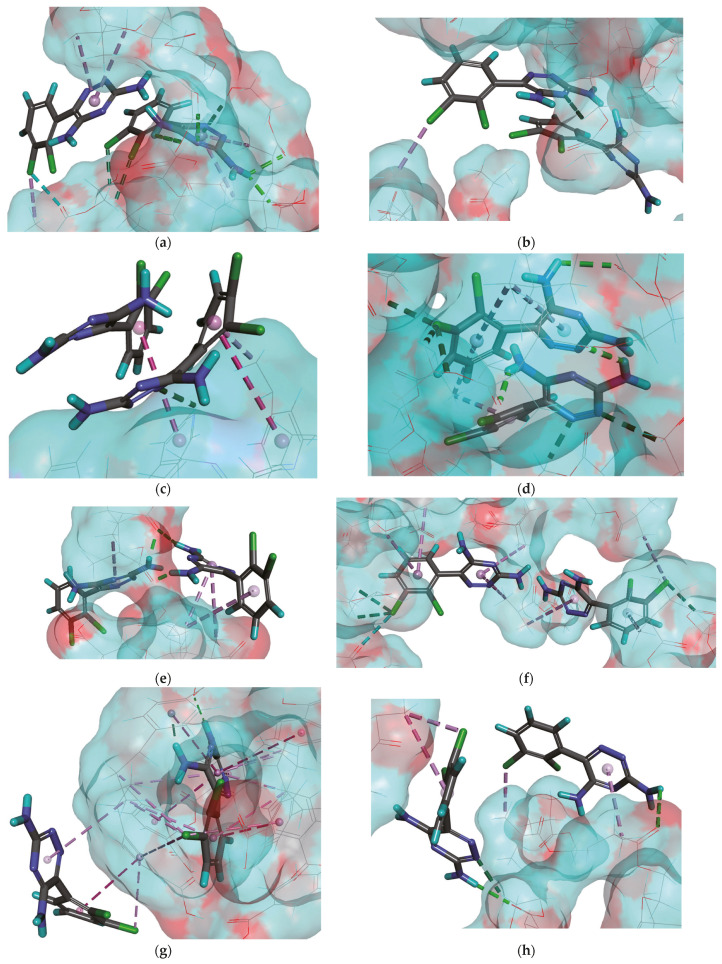

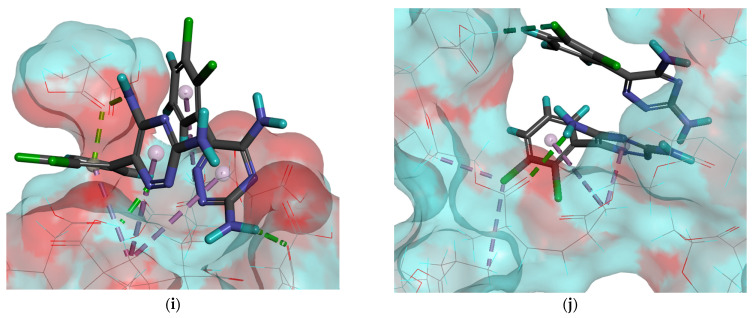
The lamotrigine molecules in the cavity of MIP**1**–MIP**5** after simulation of adsorption process. Interactions between the monomer residue and the analyte in the MIP**1** (**a**), MIP**2** (**c**), MIP**3** (**e**), MIP**4** (**g**), and MIP**5** (**i**) matrix. Interactions between the cross-linker residues and the analyte in the MIP**1** (**b**), MIP**2** (**d**), MIP**3** (**f**), MIP**4** (**h**), and MIP**5** (**j**) matrix. The classical hydrogen bonds are shown as the green dashed lines; the non-classical hydrogen bonds as the dark green dashed lines; the hydrophobic π–alkyl, alkyl–Cl, or π–Cl type interactions as the light purple dashed lines; the hydrophobic π–π stacked or T-shaped interactions as the purple dashed lines; the halogen bonds as the light blue dashed lines; and π–lone pair interaction as the green-yellow dashed line. The C atoms in molecules are represented in gray, the O atoms in red, the N atoms in blue, the H atoms in light blue, and the Cl atoms in green color.

After experimental analysis of sorption properties, binding capacity, and binding energy of the systems following lamotrigine adsorption simulation, a correlation between the values of binding capacity of polymers and the values of binding energy (Δ*E_B_*) calculated according to Equation (4) was observed (Table 1). In the MIP**1** system, which showed the lowest binding capacity and the highest binding energy (–34.8 kcal mol^−1^), we observed that both molecules of lamotrigine interacted with monomer residues from the polymeric chain (Figure 1a). The first one created four hydrogen bonds (utilizing the N or H atoms from the ring or the –NH_2_ groups, with lengths between 2.05 and 2.68 Å), three non-classical hydrogen bonds (utilizing the Cl atoms or π electrons, with lengths between 2.36 and 2.64 Å), and two hydrophobic π–alkyl type interactions (with a length of 4.30 Å). The second lamotrigine molecule formed three hydrophobic π–alkyl or alkyl–Cl type interactions (with lengths between 3.67 and 4.51 Å) and one halogen bond (utilizing the Cl atom, with a length of 2.88 Å). Additionally, the second analyte molecule interacted with cross-linker residues, forming one non-classical hydrogen bond (utilizing the N atom from the ring, with a length of 3.05 Å) and one hydrophobic alkyl–Cl type interaction (with a length of 3.69 Å, Figure 1b). Moreover, a π–π stacking interaction was observed between both lamotrigine molecules. In the MIP**2** characterized by a binding capacity value of 0.104 ± 0.007 µmol g^−1^ and a binding energy value of –149.3 kcal mol^−1^, we found that one analyte molecule interacted with monomer residues in the polymeric chain creating only one π–π stacking interaction (with a length of 5.47 Å). The second lamotrigine molecule formed one π–π stacking interaction (with a length of 5.85 Å), one hydrophobic π–alkyl type interaction (with a length of 3.62 Å), and one non-classical hydrogen bond (utilizing the N atom from the ring, with a length of 2.51 Å) with 4-vinylpyridine residues (Figure 1c). Both analyte molecules interacted with cross-linker residues in the polymeric chain (Figure 1d). The first one created two hydrogen bonds (utilizing the H atoms from the –NH_2_ groups, with lengths of 2.44 and 2.62 Å), two non-classical hydrogen bonds (utilizing the N atoms from the ring, with lengths of 2.32 and 3.03 Å), and one hydrophobic π–alkyl type interaction (with a length of 4.43 Å). The second one formed one hydrogen bond (utilizing the H atom from the –NH_2_ group, with a length of 2.62 Å), three non-classical hydrogen bonds (utilizing the Cl atom, with lengths between 2.57 and 2.80 Å), and three hydrophobic π–alkyl type interactions (with lengths between 4.77 and 5.22 Å). Additionally, two π–π stacking interactions were observed between both lamotrigine molecules. For the MIP**3** system, we obtained a binding capacity value of 0.161 ± 0.009 µmol g^−1^ and a binding energy of –252.5 kcal mol^−1^. We observed both analyte molecules interacting with the monomer residues from the polymer (Figure 1e). The first one built two interactions: one hydrogen bond (utilizing the H atom from the –NH_2_ group, with a length of 1.99 Å) and one hydrophobic π–alkyl type interaction (with a length of 3.84 Å). The second molecule created two hydrogen bonds (utilizing the H atoms from the –NH_2_ groups, with lengths of 1.95 and 2.52 Å) and three hydrophobic π–alkyl type interactions (with lengths between 4.12 and 5.12 Å). Additionally, both lamotrigine molecules formed interactions with the cross-linker residues (Figure 1f). The first one created two non-classical hydrogen bonds (utilizing the Cl atom, with lengths of 2.64 and 2.90 Å), four hydrophobic π–alkyl type interactions (with lengths between 4.07 and 5.08 Å), and one halogen bond (with a length of 3.03 Å). Between the cross-linker residues and the second lamotrigine molecule, we found one non-classical hydrogen bond (involving the Cl atom, with a length of 2.70 Å) and three hydrophobic π–alkyl type interactions (with lengths between 4.27 and 4.90 Å). Analyte molecules interacted with each other, creating one hydrogen bond. In the MIP**4** system, characterized by binding capacity and binding energy values of 0.295 ± 0.005 µmol g^−1^ and −297.2 kcal mol^−1^, respectively, we found that both molecules of lamotrigine interacted with the monomer residues (Figure 1g). The first one created one π–π stacking interaction (with a length of 4.57 Å) and two hydrophobic π–alkyl or π–Cl type interactions (with lengths of 4.33 and 4.85 Å). The second molecule formed one hydrogen bond (utilizing the H atom from the –NH_2_ group, with a length of 2.02 Å), one non-classical hydrogen bond (utilizing the H atom from the –NH_2_ group, with a length of 1.82 Å), five π–π stacking or T-shaped interactions (with lengths between 3.82 and 5.48 Å), and ten hydrophobic π–alkyl, alkyl–Cl, or π–Cl type interactions (with lengths between 3.61 and 5.01 Å). Furthermore, both molecules interacted with cross-linker residues (Figure 1h). The first one created one hydrogen bond (utilizing the H atom from the –NH_2_ group, with a length of 2.49 Å), one non-classical hydrogen bond (utilizing the N atom from the ring, with a length of 2.46 Å), and two hydrophobic π–alkyl or alkyl–Cl type interactions (with lengths between 3.25 and 4.70 Å). The second molecule formed one hydrogen bond (utilizing the H atom from the –NH_2_ group, with a length of 2.83 Å) and two hydrophobic π–alkyl or alkyl–Cl type interactions (with lengths of 3.58 and 4.13 Å). No interactions were observed between the analyte molecules. In the MIP**5** system, which showed the highest binding capacity and the lowest binding energy (Table 1), both lamotrigine molecules interacted with the monomer residues (Figure 1i). The first molecule created one hydrogen bond (utilizing the H atom from the –NH_2_ group, with a length of 2.35 Å) and two hydrophobic π–alkyl type interactions (with lengths of 5.19 and 5.24 Å), while the second molecule formed two hydrogen bonds (utilizing the N or H atoms from the ring or the –NH_2_ groups, with lengths of 2.07 and 2.72 Å), two hydrophobic π–alkyl type interactions (with lengths of 4.48 and 5.30 Å), and one π–lone pair interaction (with a length of 2.51 Å). Additionally, both lamotrigine molecules formed interactions with the cross-linker residues (Figure 1j). The first one created only one non-classical hydrogen bond (utilizing the Cl atom, with a length of 2.72 Å), and the second formed one hydrogen bond and four hydrophobic π–alkyl or π–Cl type interactions (with lengths between 3.14 and 5.36 Å). Analyte molecules interacted with each other, creating one hydrogen bond and two π–π stacking interactions.

During the analysis of the theoretical models of the MIP binding sites, we observed that in the system (MIP**5**), where the number of interactions between the analyte molecules and the monomer residues, as well as between the analyte and the cross-linker residues, were similar, the binding capacity, selectivity, and binding energy were the most favorable. We could suppose that the interactions of the analyte with the monomer are crucial and determine the specificity of the MIP, but interactions with the cross-linker also play a significant role during the creation of the binding cavity and could impact the shape of the recognition site. Considering two molecules of the analyte during simulation procedures, we found that in four systems (MIP**1**–MIP**3** and MIP**5**), lamotrigine molecules interacted with each other. In the prepolymerization complexes models, template molecules interacted with each other in the systems of MIP**1**, MIP**2**, and MIP**5**. We could suggest that interactions between the template and the analyte molecules could play a role in binding site creation, their spatial arrangement, and the adsorption process. This fact could also affect the selectivity because the MIP**4** system, where interactions between both template and both analyte molecules were not present, showed a low IF value compared to other MIP constructed from acidic monomers.

#### 2.1.3. Zeta Potential of Polymers

To provide additional information regarding the surface characteristics of the resulting bulk polymers, the zeta potentials of MIP**1**–MIP**5** and NIP**1**–NIP**5** were determined. The results are presented in Table 2.

As observed, the zeta potential values for all tested polymers were negative. Among the MIPs, the values were more negative for polymers prepared from acidic monomers (MIP**3**–MIP**5**) compared to those prepared from non-acidic monomers (MIP**1** and MIP**2**), with the lowest value recorded for MIP**5**, the polymer characterized by the highest binding capacity and specificity. Conversely, the respective NIPs, prepared from acidic monomers (NIP**3**–NIP**5**) exhibited less negative values of the zeta potential. In contrast, NIPs prepared from non-acidic monomers (NIP**1** and NIP**2**) showed lower zeta potential values compared to their molecularly imprinted counterparts. Furthermore, the results indicate that NIP**1** and NIP**2**, prepared from 2-hydroxyethyl methacrylate or 4-vinylpyridine, respectively, had lower zeta potential values compared to NIP**3**–NIP**5**, prepared from methacrylic acid, 4-vinylbenzoic acid, or itaconic acid, respectively. This suggests that the presence of monomer residues in the polymer network influences the zeta potential values of the polymers.

Additionally, we observed changes in the pH values of the solution after polymer treatment, indicating proton association–dissociation equilibrium. The most notable difference was noted for MIP**5**, with shift in pH value to 8.80. This can be explained by the association of protons from the solution to ionizable groups of the polymer matrix, particularly from the itaconic acid residues.

#### 2.1.4. Composition and Morphology of Bulk Polymers

To confirm the composition of obtained polymers, the X-ray electron dispersive spectroscopy (EDS) was employed to determine the percentage of the carbon and oxygen atoms. The results are presented in Table 3.

As observed, the presence of carbon and oxygen atoms was confirmed in all tested polymers.

The difference between polymers (MIP**1**/NIP**1**–MIP**5**/NIP**5**) was related to the presence of the residues from various monomers. It can be seen that the variations in the % weight of both atoms were more significant for MIPs (between 71 and 80 for carbon) than for NIPs (between 76 and 78 for carbon). Interestingly, in the case of MIP**2**/NIP**2**, the nitrogen atom was not detected, probably due to the insufficient sensitivity of the analysis method.

**Figure 2:**
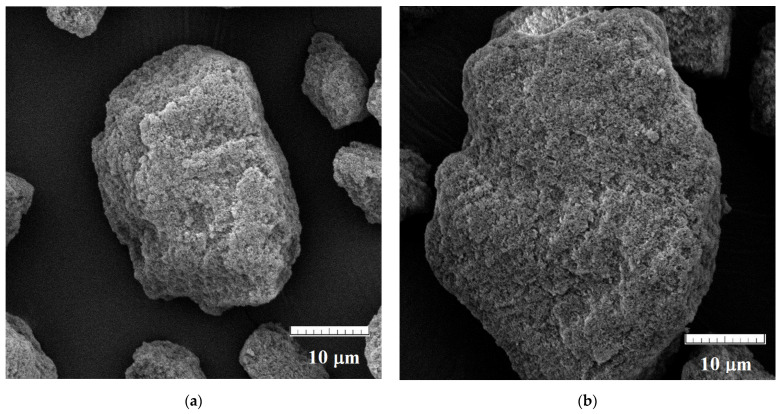
Micrographs of MIP**5** (**a**) and NIP**5** (**b**).

Finally, the morphologies of polymers were evaluated, revealing typical for bulk materials irregular size with an average diameter of 10–20 µm. All materials possessed a highly extended surface with only subtle differences between MIPs and NIPs. The exemplary micrographs of MIP**5** and NIP**5** are presented in Figure 2.

To sum up, the optimization of the synthetic process to obtain imprinted polymer towards lamotrigine was performed, revealing the highest specificity for polymer composed of itaconic acid and EGDMA. The zeta potential measurements showed significant differences between the obtained materials. The composition of polymers was confirmed, and the morphologies were evaluated, revealing only subtle variations in the extension of the surface of molecularly imprinted and non-imprinted polymers.

### 2.2. Magnetic Molecularly Imprinted Drug Carrier

#### 2.2.1. Synthesis and Adsorption Properties

In the next step of our investigations, we have decided to fabricate a magnetic molecularly imprinted carrier for lamotrigine. Taking into account the application of the material as the drug carrier, the additional properties of the carrier, such as response to the external magnetic field, could facilitate its positioning in the specific location of the body. Moreover, magnetic core-shell materials are characterized by well-defined morphology, and the presence of a molecularly imprinted thin shell layer facilitates the sorption behavior of the molecularly imprinted material. For that purpose, the previously optimized composition of the MIP was used for surface modification of functionalized magnetite, resulting in the fabrication of the MIP**5-mag** material. The non-imprinted polymer, coded as NIP**5-mag** was also synthesized.

The binding capacities (*B*, ng g^−1^) were determined, and selectivity was calculated according to Equations (1)–(3). The results are presented in Table 4.

As it can be seen, the binding capacity of MIP**5-mag** was more than three-fold higher when compared to NIP**5-mag**, confirming the high specificity of MIP**5-mag** material.

#### 2.2.2. Composition and Morphology of Core-Shell Materials

In order to analyze the composition of the MIP**5-mag** and NIP**5-mag**, the EDS measurements were carried out to determine the percentage of carbon, oxygen, silicon, and iron.

**Figure 3:**
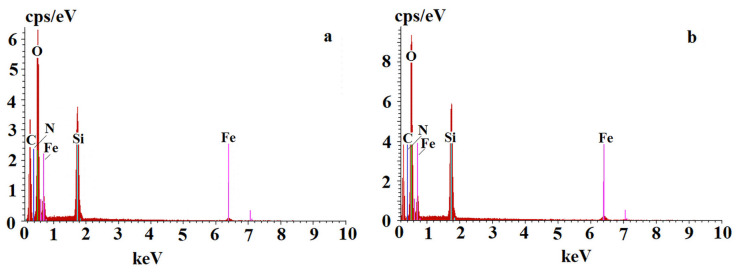
EDS spectra for MIP**5-mag** (**a**) and NIP**5-mag** (**b**).

The results were as follows (% weight), for MIP**5-mag**: C, 25 ± 2, O, 39 ± 2, Si, 19 ± 1, Fe, 15 ± 1 and for NIP**5-mag**: C, 18 ± 4, O, 38 ± 1, Si, 24 ± 4, Fe, 19 ± 2, respectively. The EDS spectra for MIP**5-mag** and NIP**5-mag** are presented in Figure 3. The results confirmed the presence of magnetite in the MIP**5-mag** and NIP**5-mag** together with functionalized layer of siloxane and organic polymer.

Next, the Fourier transform infrared (FT-IR) analysis was carried out to confirm the structure of MIP**5-mag** and NIP**5-mag**.

**Figure 4:**
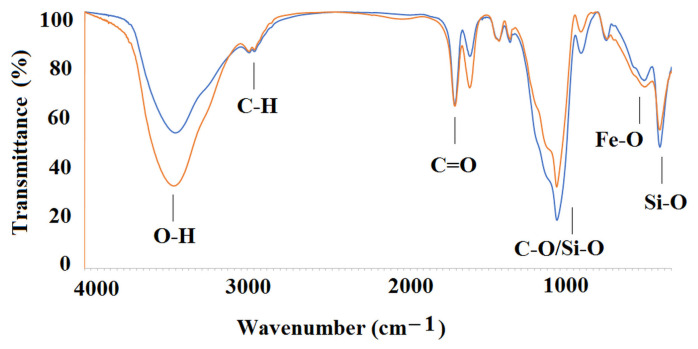
FT-IR spectra of MIP**5-mag** (red line) and NIP**5-mag** (blue line).

Figure 4 presents the spectra of MIP**5-mag**/NIP**5-mag** and Table 5 presents the values of vibrations of main functional groups from MIP**5-mag** and NIP**5-mag** as well as, for the comparison, the values of vibrations of main functional groups from neat cross-linker.

As it can be seen, the spectra of MIP**5-mag** and NIP**5-mag** revealed characteristic peak that is attributed to the Fe–O vibrations, peaks that could be assigned to Si–O, and peaks from various C–H, C=O or C–O vibrations, confirming the structure of materials.

**Figure 5:**
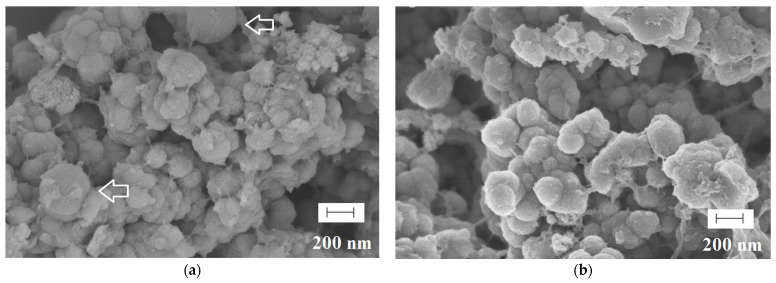
Micrographs of MIP**5-mag** (**a**) and NIP**5-mag** (**b**).

Finally, the morphology of MIP**5-mag** and NIP**5-mag** was studied using scanning electron microscopy (SEM). The micrographs are presented in Figure 5.

As it can be seen, the spherical particles of MIP**5-mag** (a) and NIP**5-mag** were obtained. The magnetic particles for both materials are characterized by the diameter of around 100 nm. The organic polymer conjugations could be found between these particles confirming that the polymerization process occurred. In Figure 5a (see arrows), regular particles of higher diameter (around 200 nm) can be found. It could be supposed that neat siloxane particles were also formed simultaneously. This phenomenon is known but it could affect the homogeneity of the material [37].

#### 2.2.3. Crystal Structure and Magnetic Properties

**Figure 6:**
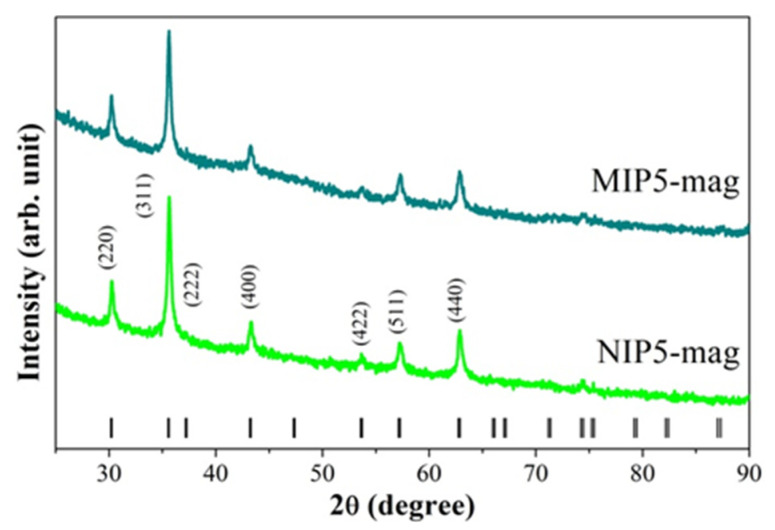
The X-ray diffraction patterns of MIP**5-mag** and NIP**5-mag** samples obtained at room temperature. The ticks positioned along the bottom of the graph correspond to the theoretical locations of Bragg peaks associated with the cubic magnetite structure (*Fd*-3*m*). The most prominent peaks were characterized using Miller indices (*hkl*).

To verify the quality of the magnetite-based samples, we performed X-ray diffraction (XRD) measurements. Diffractograms measured at room temperature for the MIP**5-mag** and NIP**5-mag** samples are presented in Figure 6. Analyzing the XRD patterns, we observed only peaks originating from Fe_3_O_4_, which crystallizes in a cubic structure (space group *Fd*-3*m*, no. 227). The average crystallite size, which we determined from the Scherrer equation [38], was about 20 nm for both samples. The XRD measurements confirmed the presence of nanocrystalline Fe_3_O_4_ in the magnetic core.

**Figure 7:**
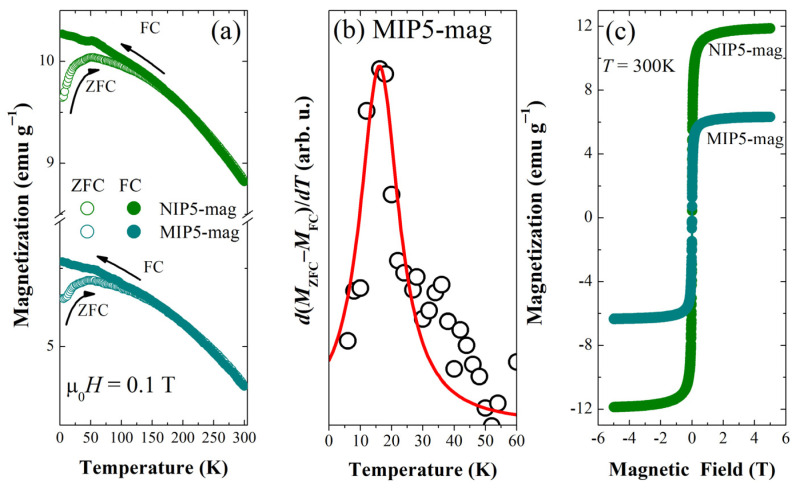
Magnetic properties of MIP**5-mag** and NIP**5-mag** samples. (**a**) Temperature dependence of magnetization measured in a magnetic field of 0.1 T. ZFC—zero-field cooling, FC—field cooling. (**b**) Determination of the blocking temperature for the MIP**5-mag** sample from the *d*(*M*_ZFC_ − *M*_FC_)/*dT* relationship. The solid red line represents Lorentz fit. (**c**) Magnetization curves were measured at a constant temperature of 300 K.

To determine the magnetic properties of the MIP**5-mag** and NIP**5-mag** samples, we made a series of measurements using a vibrating sample magnetometer (VSM). The results of the measurements and their analysis are collected in Figure 7. The temperature dependence of the magnetization *M*(*T*) in a constant magnetic field μ_0_*H* = 0.1 T measured in zero-field cooling (ZFC) and field cooling (FC) mode has a similar shape for both samples and is typical of low-dimensional magnetite. The magnetization curves successively increase with increasing temperature for both ZFC and FC curves. At around 150 K they split, after which the FC curve continues to rise, while for the ZFC curve, a broad maximum appears at ~50 K and its value begins to decrease with further decrease in temperature (Figure 7a). The *M*(*T*) relation does not show an anomaly in the vicinity of 120 K, which could be related to the Verwey transition, typical for Fe_3_O_4_ [39]. By plotting the *d*(*M*_ZFC_ − *M*_FC_)/*dT* dependence, the blocking temperature *T*_B_ of superparamagnetic particles was determined. The value of *T*_B_ was determined by fitting the *d*(*M*_ZFC_ − *M*_FC_)/*dT*(*T*) relation using the Lorentz function (Figure 7b) [40]. The *T*_B_ values obtained by this method are about 16 K for MIP**5-mag** and NIP**5-mag** samples.

The magnetization curves *M*(μ_0_*H*) were measured at *T* = 300 K and 4 K in a magnetic field up to 5 T. Both samples showed behavior typical of superparamagnetic materials (Figure 7c). Magnetic hysteresis was not observed at room temperature for these materials, but at *T* = 4 K, a noticeable hysteresis loop with a coercivity field of approximately 20 mT was detected. The maximum value of magnetization at *T* = 300 K and for μ_0_*H* = 5 T was 6.33 ± 0.01 emu g^−1^ and 11.87 ± 0.01 emu g^−1^ for MIP**5-mag** and NIP**5-mag**, respectively. The reduced *M* value observed in the MIP**5-mag** sample was a result of its lower magnetite content in comparison to the other non-magnetic constituents, especially when contrasted with the NIP**5-mag** sample. In the NIP**5-mag** sample, due to the absence of polymer, the mass ratio of magnetic material to the remaining non-magnetic components was higher, leading to a higher magnetization value determined for the entire sample mass.

### 2.3. Lamotrigine Desorption and Release Study

At the last stage of our investigations, we analyzed desorption of lamotrigine at different pH values of phospahe buffer saline (PBS) and investigated the release profiles. Lamotrigine was adsorbed to MIP**5-mag** and NIP**5-mag** at the concentration of 100 μg L^−1^ in methanol–water solution (85:15 *v*/*v*) to ensure sufficient binding capacity and to maintain the specificity as high as possible. The binding capacities were as follows: 3.4 ± 0.7 μg g^−1^ for MIP**5-mag** and 3.3 ± 0.3 μg g^−1^ for NIP**5-mag**, respectively. The desorption and release profiles of lamotrigine were analyzed in the PBS as a release medium adjusted to pH 2 or pH 5 or pH 8.

Firstly, we analyzed the percentage of the adsorbed lamotrigine amount that was released from MIP**5-mag** and NIP**5-mag** to PBS at pH 2, pH 5, and pH 8.

The highest percentage of adsorbed lamotrigine was released from MIP**5-mag** and NIP**5-mag** to PBS at pH 2. The total percentage release from MIP**5-mag** and NIP**5-mag** after 6 h of the experiment was equal to 59.52% and 60.99%, respectively. On the contrary, the total percentages of lamotrigine release after 6 h of experiment to PBS at pH 5 and at pH 8 were significantly lower with the following values for pH 5: 23.40% and 19.06% for MIP**5-mag** and NIP**5-mag**, respectively, and for pH 8: 19.86% and 17.70% for MIP**5-mag** and NIP**5-mag**, respectively.

Next, we analyzed the cumulative amount of lamotrigine released from MIP**5-mag** and NIP**5-mag** as a function of time.

**Figure 8:**
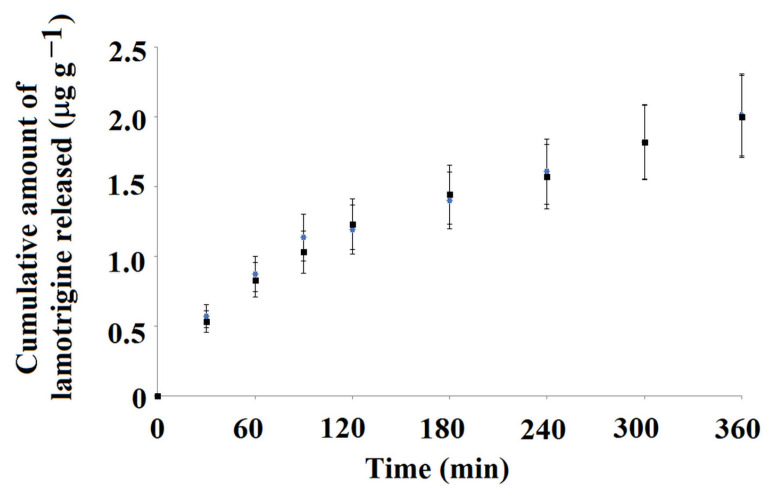
Cumulative amount of lamotrigine released from MIP**5-mag** (blue) and NIP**5-mag** (black) to PBS adjusted to pH 2 as a function of time, *n* = 3.

Figure 8 presents data for the cumulative amount of lamotrigine that was released to PBS at pH 2.

The release profile of lamotrigine to PBS at pH 2 was very similar for both tested materials, viz. MIP**5-mag** and NIP**5-mag**. The release amount of lamotrigine until the 2 h of experiment was characterized by higher values, and then the release amount was lowered and constant in time. The higher release at the beginning of the experiment can be explained by the burst effect of lamotrigine. The cumulative values released from the MIP**5-mag** and NIP**5-mag** were as follows: 2.016 μg g^−1^ and 2.004 μg g^−1^, respectively. It could be concluded that the release profile of lamotrigine from MIP**5-mag** and NIP**5-mag** to PBS at pH 2 was very similar, and the release amounts were indistinguishable. Moreover, the pH of the release medium caused the burst effect.

**Figure 9:**
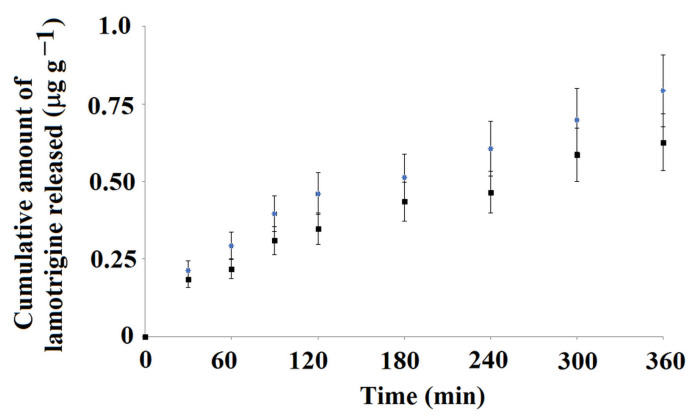
Cumulative amount of lamotrigine released from MIP**5-mag** (blue) and NIP**5-mag** (black) to PBS adjusted to pH 5 as a function of time, *n* = 3.

Figure 9 presents data for the cumulative amount of lamotrigine that was released to PBS at pH 5.

**Figure 10:**
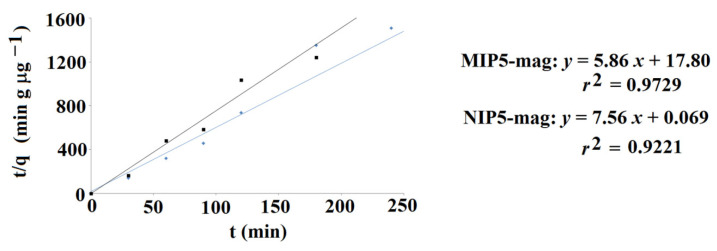
Pseudo-second order kinetics of lamotrigine to PBS at pH 5 for MIP**5-mag** (blue) and NIP**5-mag** (black).

The release profile of lamotrigine to PBS at pH 5 was different when compared to release to PBS at pH 2. The total release amounts were lower and burst effect was not observed. Moreover, the cumulative amounts of lamotrigine released from the MIP**5-mag** and NIP**5-mag** differed as follows: 0.793 μg g^−1^ and 0.626 μg g^−1^, respectively. It could be concluded that the release profile of lamotrigine from MIP**5-mag** and NIP**5-mag** to PBS at pH 5 was different, with the higher cumulative amount of lamotrigine released from molecularly imprinted material. In order to emphasize the difference, the desorption kinetics were investigated. Data were fitted into the mathematical model (Equation (5)). The results confirmed that the pseudo-second order kinetics governed the desorption process (Figure 10). The calculated values of the *q*_e_ and *K*_2_ constants were as follows: *q*_e_ = 0.171 μg g^−1^ and *K*_2_ = 1.93 g (μg min)^−1^ for MIP**5-mag** and *q*_e_ = 0.132 μg g^−1^ and *K*_2_ = 577 g (μg min)^−1^ for NIP**5-mag**.

**Figure 11:**
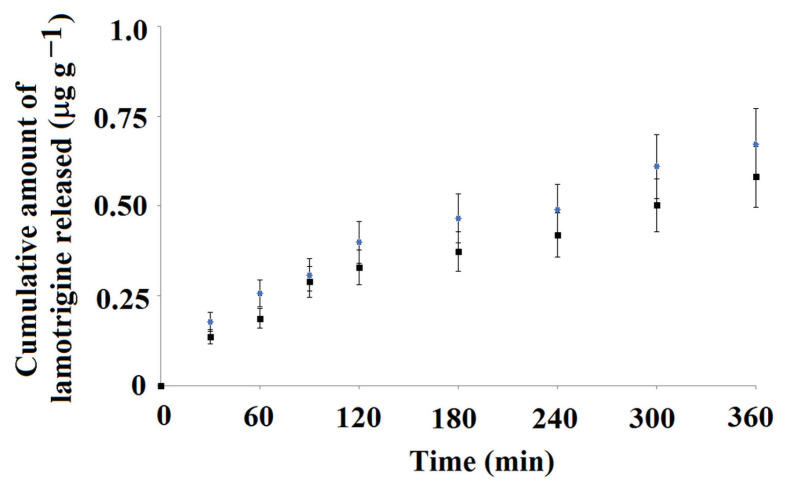
Cumulative amount of lamotrigine released from MIP**5-mag** (blue) and NIP**5-mag** (black) to PBS adjusted to pH 8 as a function of time.

Figure 11 presents data for the cumulative amount of lamotrigine that was released to PBS at pH 8.

**Figure 12:**
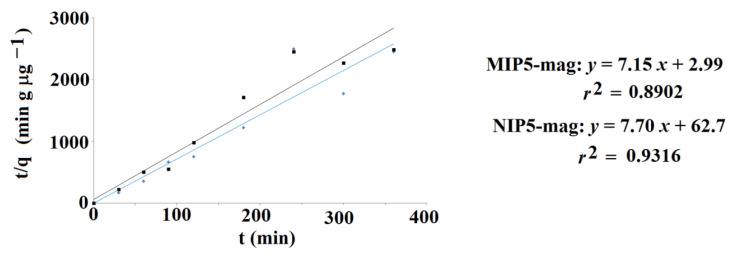
Pseudo-second order kinetics of lamotrigine to PBS at pH 8 for MIP**5-mag** (blue) and NIP**5-mag** (black).

The release profile of lamotrigine to PBS at pH 8 was similar when compared to release to PBS at pH 5. The total amounts of lamotrigine released from the material were a little lower when compared to the release at pH 5, and, once again, burst effect was not observed. The cumulative amounts of lamotrigine released from the MIP**5-mag** and NIP**5-mag** differed as follows: 0.673 μg g^−1^ and 0.582 μg g^−1^, respectively. To analyze the data, the desorption kinetics were investigated. Data were fitted into the mathematical model (Equation (5)). The results confirmed that the pseudo-second order kinetics governed the desorption process (Figure 12). The calculated values of the *q*_e_ and *K*_2_ constants were as follows: *q*_e_ = 0.140 μg g^−1^ and *K*_2_ = 17.1 g (μg min)^−1^ for MIP**5-mag** and *q*_e_ = 0.130 μg g^−1^ and *K*_2_ = 0.945 g (μg min)^−1^ for NIP**5-mag**.

Similar trends of drug release from the MIP matrix according to pH dependence were observed; for example, for tramadol release from the MIP build from methacrylic acid and EGDMA [41], for matrine release from MIP constructed from methacrylic acid and dopamine [42], and for metronidazole release from MIP formed from itaconic acid and EGDMA [43]. All above mentioned chemical compounds possess basic character and their solubility increases in lower pH values. This fact causes an acceleration in the release of the compound into more acidic media. Additionally, protonation of acidic group in the MIP matrix affects the interaction between the drug and MIP. An opposite pH effect on drug release from the MIP matrix was observed for acidic compounds and the MIP matrix build from basic monomers, as, for example, for ibuprofen release from MIP build from 2-(dimethylamino)ethyl methacrylate and EGDMA [44] or for diclofenac release from MIP formed from 4-vinylpyridine [45].

To sum up, the release profiles of lamotrigine from the MIP**5-mag** and NIP**5-mag** to PBS adjusted to pH 2, pH 5, or pH 8 were different. The highest percentage of adsorbed lamotrigine was released to PBS at pH 2 with no difference between imprinted and non-imprinted materials and with significant burst effect. In contrast, the release profiles of lamotrigine to PBS at pH 5 or at pH 8 were characterized by lower percentages of adsorbed lamotrigine released. Comparing cumulative amounts of lamotrigine released from imprinted and non-imprinted materials at pH 5 and pH 8, higher values were observed for MIP**5-mag**. Desorption analyses revealed that pseudo-second order kinetics governed the desorption from MIP**5-mag** and NIP**5-mag**.

## 3. Materials and Methods

### 3.1. Reagents and Standards

Template molecule, 2,4-diamine-1,3,5-triazine, and the analyte, lamotrigine were purchased from Tokyo Chemical Industry (Tokyo, Japan). The functional monomers: 2-hydroxyethyl methacrylate (**1**), 4-vinylpyridine (**2**), methacrylic acid (**3**), 4-vinylbenzoic acid (**4**), and itaconic acid (**5**) were from Sigma-Aldrich (Steinheim, Germany). The cross-linker, EGDMA, tetraethoxysilane, and 3-(trimethoxysilyl) propyl methacrylate (MPS) were purchased from Sigma-Aldrich (Steinheim, Germany). The 2,2′-azobis(2-methylproprionitrile), the initiator, was from Merck (Darmstadt, Germany). Trisodium citrate dehydrate, sodium hydroxide, sodium nitrate, ammonium acetate, acetonitrile, methanol, ethanol, toluene, and acetone were obtained from POCH (Gliwice, Poland). Ferrous sulphate heptahydrate was delivered by Avantor Performance Materials (Gliwice, Poland), dimethyl sulfoxide and PBS were bought from Sigma-Aldrich (Steinheim, Germany). Ammonium hydroxide was purchased from Chempur (Piekary Śląskie, Poland). Internal standard, lamotrigine-*d*_3_ was purchased from Tokyo Chemical Industry (Tokyo, Japan). The HPLC gradient-grade solvents: methanol, acetonitrile, and formic acid 98% were purchased from Merck (Darmstadt, Germany). Ultrapure water delivered from a Hydrolab HLP 5 system (Straszyn, Poland) was used to prepare the water solutions.

### 3.2. Polymers

#### 3.2.1. Bulk Polymers

The bulk thermal radical polymerization process was used for optimization of the composition of most effective material. The bulk MIPs coded as MIP**1**–MIP**5** were fabricated in the presence of 2,4-diamine-1,3,5-triazine (template molecule) together with non-imprinted polymers, coded as NIP**1**–NIP**5** that were fabricated under the same polymerization conditions but without the template molecule. The experimental amounts of the reagents (moles, masses, and volumes) used for the preparation of the different types of polymers are listed in Table 6.

The bulk polymerization was performed according to our previous work [46]. Details are provided in the Appendix A.1.

#### 3.2.2. Magnetic Core-Shell Material

The core-shell magnetic nanoconjugates were fabricated for the analytical measurements using MIP shell, optimized in a previous step. The magnetic core was prepared as described previously [47] prior to the functionalization by a silane derivative, providing the functional groups and enabling the polymerization of the imprinted layer on its surface. Details are provided in the Appendix A.2.

### 3.3. Instruments

Instrumental analysis was carried out for magnetic core-shell material using an Agilent 1260 Infinity System (Agilent Technologies, Santa Clara, CA, USA), equipped with a degasser, an autosampler, and a binary pump, coupled to a QTRAP 4000 hybrid triple quadrupole/linear ion trap mass spectrometer (AB Sciex, Framingham, MA, USA). Details of the operational parameters are provided in the Appendix A.3.

The dynamic light scattering analyses of polymers were measured using Malvern Zetasizer (Worcestershire, UK).

The micro-chemical and surface morphology analysis was performed using SEM with a Model TS VEGA LMU Scanning Electron Microscope (Tescan, Brno, Czech Republic) equipped with an EDS INCA Penta FET X3 system.

Powder XRD patterns were acquired on an X’Pert Pro diffractometer (PANalytical, Almelo, The Netherlands) in Bragg–Brentano geometry with a Ni-filtered CuKα radiation (λ = 1.5418 Å) and a PIXcel solid-state linear detector, at 40 kV and 30 mA current. Experiments were carried out in a 2θ angular range of 20 to 90° with a step size of 0.026° and a total scan time of 30 min. Data for both powder samples were collected at room temperature.

Magnetic properties were verified by measuring magnetization as a function of temperature and external magnetic field strength using VSM, which is an option of the Physical Properties Measurement System (PPMS) by Quantum Design (San Diego, CA, USA).

### 3.4. Adsorption Tests

The stationary binding experiments were performed to evaluate the binding ability of MIPs and NIPs towards lamotrigine. Details of the adsorption tests are provided in the Appendix A.4.

On the basis of the adsorption measurements, the parameters that characterize the polymers were calculated according to Equations (1)–(3). The binding capacities (*B*) of MIPs and NIPs were calculated according to Equation (1):

(1) $$B=\frac{(C_{i}-C_{f})V}{m}$$

followed by the distribution coefficients (*K_D_*) for MIPs and NIPs that were calculated according to Equation (2):

(2) $$K_{D}=\frac{(C_{i}-C_{f})V}{\;C_{f}m}$$

where *V* represents the volume of solution (L), *C_i_* represents the initial solution concentration, *C_f_* represents the solution concentration after adsorption, and *m* is the mass of particles (g). Then, the imprinting factors (IF) were calculated according to Equation (3):

(3) $$\mathrm{IF}=\frac{K_{D}(\mathrm{MIP})}{K_{D}\;(\mathrm{NIP})}$$

### 3.5. Theoretical Analysis

Molecular modelling methodology was performed according to our previous works [46,48]. Firstly, the optimization of all compound structures together with the calculation of so-called ESP (electrostatic potential) charges was performed using the density functional theory with a B3LYP/6–311+G(d,p) hybrid functional, implemented in the Gaussian 16 program [49]. The Packmol software (version 18.169) [50] was applied to obtain random starting models of analyzed systems. A CHARMM force field [51], the Leapfrog Verlet integration, and SHAKE [52] algorithms were employed to parametrize all molecular systems and simulation processions during molecular mechanics (MM) and molecular dynamic (MD) simulations performed by BIOVIA Discovery Studio 2022 software package [53]. Details are provided in the Appendix A.5.

The binding energies (Δ*E_B_*, kcal mol^−1^) were calculated according to Equation (4):

∆*E_B_* = *E_system_* − 2*E_analyte_* − *E_cavity_* (4)

where *E_system_* is the potential energy of the MIP cavity with bound analyte in the solvent, *E_analyt_*_e_ is the potential energy of lamotrigine, and *E_cavity_* is the potential energy of the MIP cavity without the analyte in the solvent.

### 3.6. Release Studies

Twenty milligrams of MIP5**-mag** or NIP**5-mag** were weighed into test tubes. Then, a volume of 1 mL of lamotrigine at the concentration of 100 μg L^−1^ in methanol–water solution (85:15 *v*/*v*) was added and placed on a shaker at room temperature for 24 h. Afterwards, the supernatant was discarded, and the pellet was washed for 1 min with water (1 mL). Finally, the MIP**5-mag** or NIP**5-mag** were incubated in PBS solutions at pH 2.0, pH 5.0, and pH 8.0 on a shaker. Samples of a volume of 500 µL were collected at 0.5, 1.0, 1.5, 2.0, 3.0, 4.0, 5.0, and 6.0 h, and the collected solutions were replaced with fresh PBS adjusted to the appropriate pH value. The collected samples were then mixed with an internal standard (9:1, *v*/*v*, 1 mg L^−1^) and injected into the HPLC system. Instead of centrifugation to separate the polymer from the liquid phase, a magnetic field was applied.

The desorption kinetics were analyzed using the pseudo-second-order equation as shown in Equation (5):

(5) $$\frac{t}{q}=\frac{1}{k2qe^{2}}+(\frac{1}{qe})t$$

where *k*_2_ is the second-order-rate constant at the equilibrium (*q_e_*).

## 4. Conclusions

Theoretical modeling of the MIP binding sites revealed that in the systems where the interactions between analyte molecules and monomer residues, as well as between analyte and cross-linker molecules, were similar, the binding capacity, selectivity, and binding energy were most favorable. These in silico analyses were corroborated by the examination of the binding properties of molecularly imprinted poly(itaconic acid-co-ethylene glycol dimethacrylate) material. Additionally, the structure and magnetic behavior of the lamotrigine drug carrier were characterized, indicating the superparamagnetic properties of the analyzed material. Notably, the release profiles of lamotrigine into PBS adjusted to pH 2, pH 5, or pH 8 differed. The magnetic MIP exhibited controlled drug release without a burst effect in PBS at pH 5 and 8. These findings hold significant potential for the development of a lamotrigine drug delivery system for administration via nasal route.

## Figures and Tables

**Table 1:** Values of binding capacities of lamotrigine on MIP**1**–MIP**5** and NIP**1**–NIP**5** (conc. 1 μmol L^−1^, *n* = 3), distribution ratios *K*_D_, IFs, and binding energy ∆*E_B_*.

No of Polymer	Binding Capacities ± S.D. (*B*, µmol g^−1^)		Distribution Ratio (*K*_D_, L g^−1^)		IF	Binding Energy (∆*E_B_*, kcal mol^−1^)
	MIP	NIP	MIP	NIP		
**1**	0.089 ± 0.003	0.20 ± 0.02	0.087	0.258	0.34	−34.8
**2**	0.104 ± 0.007	0.083 ± 0.001	0.104	0.080	1.30	−149.3
**3**	0.161 ± 0.009	0.106 ± 0.004	0.184	0.108	1.70	−252.5
**4**	0.295 ± 0.005	0.29 ± 0.01	0.483	0.453	1.07	−297.2
**5**	0.32 ± 0.01	0.089 ± 0.003	0.574	0.087	6.62	−317.7

**Table 2:** Zeta potential and the pH values of samples MIP**1**–MIP**5** and NIP**1**–NIP**5**.

No of Polymer	Zeta Potential (*ζ*, mV)		Sample pH * (at 25 °C)	
	MIP	NIP	MIP	NIP
**1**	−1.01	−53.7	8.13	7.58
**2**	−11.8	−40.4	7.72	8.77
**3**	−41.5	−26.6	7.32	8.54
**4**	−29.2	−0.932	7.53	7.10
**5**	−56.5	−39.3	8.80	8.29

* pH of the solution was set at 6.79.

**Table 3:** The percentage of C and O atoms in samples MIP**1**–MIP**5** and NIP**1**–NIP**5** (*n* = 3).

No of Polymer	C, % Weight		O, % Weight	
	MIP	NIP	MIP	NIP
**1**	76 ± 5	77 ± 3	24 ± 5	23 ± 3
**2**	80 ± 4	78 ± 3	20 ± 4	23 ± 3
**3**	76 ± 1	76 ± 2	24 ± 1	24 ± 2
**4**	71 ± 1	77 ± 5	28 ± 1	23 ± 5
**5**	74 ± 5	76 ± 4	26 ± 5	24 ± 4

**Table 4:** Binding capacities of lamotrigine on MIP**5-mag** and NIP**5-mag** (conc. 50 μg L^−1^, *n* = 3), distribution ratios *K*_D_ and IFs.

No of Polymer	Binding Capacities ± S.D. (*B*, ng g^−1^)		Distribution Ratio (*K*_D_, L g^−1^)		IF
	MIP	NIP	MIP	NIP	
**5-mag**	984 ± 27	316 ± 23	0.0258	0.0070	3.66

**Table 5:** The values of vibrations of main functional groups from MIP**5-mag**/NIP**5-mag** and from cross-linker.

Material/Compound	MIP5-mag	NIP5-mag	EGDMA
Bond	Vibration (cm^−1^)		
O–H	3417 (broad)	3417 (broad)	-
C–H	2962, 2946	2962, 2946	2963, 2932
C=O	1728	1728	1726
C–O	1338-981 (broad) *	1340-1010 (broad) *	1299, 1162 (sharp)
Si–O	1338-981 (broad) *	1340-1010 (broad) *	-
Fe–O	530	530	-
Si–O	470	470	-

* vibrations overlapped.

**Table 6:** Amounts of monomers used for the polymerization of 754 μL (4 mmol) of EGDMA in the presence of 22.2 mg (0.2 mmol) 2,4-diamine-1,3,5-triazine and 10 mg of 2,2′-azobis(2-methylproprionitrile) in a mixture of 1.320 mL of dimethyl sulfoxide and 0.188 mL of acetonitrile.

Code	Functional Monomer (mg, mmol)
MIP**1**	2-Hydroxyethyl methacrylate (**1**), 104.1, 0.8
MIP**2**	4-Vinylpyridine (**2**), 84.1, 0.8
MIP**3**	Methacrylic acid (**3**), 68.9, 0.8
MIP**4**	4-Vinylbenzoic acid (**4**), 118.5, 0.8
MIP**5**	Itaconic acid (**5**), 104.08, 0.8
MIP**5-mag** *	

* It was prepared as Fe_3_O_4_@SiO_2_-MPS@MIP in a volume of 7 mL of dimethyl sulfoxide and 13 mL of acetonitrile, 22.2 mg (0.2 mmol) of 2,4-diamine-1,3,5-triazine and 104.8 mg (0.8 mmol) of itaconic acid, 754 µL (4 mmol) of EGDMA, 20 mg of 2,2′-azobis(2-methylpropionitrile), and 200 mg of Fe_3_O_4_@SiO_2_-MPS.

## Data Availability

Data will be made available on request.

## Appendix A

### Appendix A.1. Bulk Polymerization

Briefly, 2,4-diamine-1,3,5-triazine (template), the appropriate functional monomer, and EGDMA (cross-linker) were dissolved in a porogenic mixture of 1.320 mL of dimethylsulfoxide and 0.188 mL of acetonitrile in a thick-walled glass tube. A molar ratio of the template to the functional monomer and the cross-linker was equal to 1:4:20. At the end, the initiator of polymerization, 2,2′-azobis(2-methylproprionitrile) was added, and then the glass tubes were sealed. Subsequently, the polymerization was carried out under a nitrogen atmosphere for 24 h at 64 °C. The bulk rigid polymers were ground in a mortar with a pestle and wet-sieved into the particles below 45 μm diameter. Fine particles were separated by repeated decantation in acetone. The template was removed from the polymer with continuous extraction in a Soxhlet apparatus (24–36 h, 80 mL, methanol), followed by a washing sequence with 0.04 M aq. ammonium acetate–methanol (30:70 *v*/*v*) and methanol. The template removal was monitored by LC–MS analysis.

### Appendix A.2. Preparation of Magnetic Core-Shell Materials

The Fe_3_O_4_ magnetic nanoparticles were synthetized by the addition of 2.94 g (10 mmol) of trisodium citrate dehydrate, 1.63 g (40 mmol) of sodium hydroxide and 34.2 g (400 mmol) of sodium nitrate, which were dissolved in a volume of 180 mL of ultrapure water. The reaction was carried out in a round-bottom flask equipped with reflux and placed on a stirrer with a heating plate (Heidolph, Schwabach, Germany). The mixture was stirred and heated to 100 °C until a clear solution was observed. A volume of 20 mL of 1 mol L^−1^ aqueous ferrous sulphate heptahydrate solution was added and was maintained at 100 °C for one hour. After letting it cool down slowly to room temperature, a black precipitate was observed. It was separated by a magnet (discarding the solution), and then, the particles were washed with ultrapure water several times (20 mL each). Then, the obtained Fe_3_O_4_ magnetic nanoparticles were dried at room temperature prior to the modification with tetraethoxysilane to obtain Fe_3_O_4_@SiO_2_. An amount of 600 mg of Fe_3_O_4_ was suspended in 80 mL of ethanol and 8 mL of ultrapure water under ultrasonication (Bandelin, Berlin, Germany) for 15 min in a round-bottom flask. After that, a volume of 10 mL of ammonium hydroxide and a volume of 4 mL of tetraethoxysilane were added. The reaction was carried out for 12 h at room temperature with stirring. The product was separated with a magnet and was washed with ultrapure water and ethanol (20 mL each). Afterwards, the reaction of the Fe_3_O_4_@SiO_2_ with MPS was carried out to provide Fe_3_O_4_@SiO_2_-MPS. Approximately 500 mg of Fe_3_O_4_@SiO_2_ was suspended in 100 mL of anhydrous toluene and 10 mL of MPS in a three-neck round-bottom flask. The mixture was allowed to react for 12 h under nitrogen with stirring. The Fe_3_O_4_@SiO_2_-MPS was separated again with a magnet and washed with ultrapure water and ethanol (20 mL each). The prepared particles were left in dry conditions for further synthesis of a molecularly imprinted shell. The magnetic core-shell polymerization process was proceeded to prepare molecularly imprinted Fe_3_O_4_@SiO_2_-MPS@MIP (Table 6, coded as MIP**5-mag**). Briefly, to a mixture of 7 mL of dimethyl sulfoxide and 13 mL of acetonitrile, 22.2 mg (0.2 mmol) of 2,4-diamine-1,3,5-triazine and 104.8 mg (0.8 mmol) of itaconic acid were added followed by addition of a volume of 754 µL (4 mmol) of EGDMA, 20 mg of 2,2′-azobis(2-methylpropionitrile), and 200 mg of Fe_3_O_4_@SiO_2_-MPS. The mixture was sonicated for 15 min, purged with nitrogen for 5 min and left heated to 80 °C on the magnetic stirrer for 8 h to proceed polymerization process. Next, the polymer was separated and washed (using an external magnet) in the following sequence: methanol (2 × 20 mL), 40 mmol L^−1^ aqueous ammonium acetate–methanol 30:70 *v*/*v* (2 × 20 mL), and methanol (4 × 20 mL). The template molecule was extracted using the Soxhlet apparatus, lasting 36 h (120 mL of methanol) and was monitored by LC–MS.

### Appendix A.3. Details of the Operational Parameters

The turbo ion spray source was operated in positive mode. The curtain gas, ion source gas 1 and ion source gas 2, were set at 241 kPa, 414 kPa, 276 kPa and “high” instrument units (4.6 × 10^−5^ Torr), respectively. The ion spray voltage and source temperature were 5500 V and 600 °C, respectively. The target compounds were analyzed in multiple reaction monitoring (MRM) mode. The quantitative MRM transitions, declustering potential (DP) and collision energy (CE) were as follows *m*/*z* 256/211 (DP = 101 V, CE = 37 V and *m*/*z* 259/214 (DP = 51 V, CE = 37 V) for the internal standard. Chromatographic separation was achieved with a BionaCore C18 Column (100 mm × 4.6 mm, 2.7 µm) from Bionacom (Coventry, UK). The column was maintained at 40 °C at a flow rate of 0.5 mL min^−1^. The mobile phases consisted of water with 0.2% formic acid as eluent A, and acetonitrile with 0.2% formic acid as eluent B. The gradient (%B) was as follows: 0 min. 10%, 1 min. 10%, 6 min. 90% and 8 min. 90%. The re-equilibration of the column to the initial conditions lasted for 2 min. The injection volume was 10 µL.

### Appendix A.4. Details of the Adsorption Tests

Polypropylene tubes of a volume of 10 mL were filled with 10 mg of MIP**1**–MIP**5** or NIP**1**–NIP**5** particles. To each tube, a volume of 5 mL of 1 μmol L^−1^ of methanol–water (85:15 *v*/*v*) standard solution of lamotrigine was added. The tubes were sealed and oscillated by a shaker at room temperature for 3 h. Then, the tubes were centrifuged (20 min, 6000 rpm), and the aliquots of supernatant were used to analyze the unbound amounts of the compound. For the analysis of MIP**5-mag** and NIP**5-mag**, to each Eppendorf test tube, a volume of 1 mL of the standard solution of lamotrigine (c = 100 μg L^−1^) was added. The tubes were sealed and oscillated by a shaker at room temperature for 3 h. Then the external magnet was used to facilitate the separation, and the aliquots of supernatant were used to analyze the unbound amounts of the compound. The amounts of analyte bound to the polymer were calculated by subtracting the unbound amount from the initial amount. All measurements were carried out in triplicate.

### Appendix A.5. Molecular Modeling

In the first step, computational modelling involved the energy-minimization of the starting models resulted by Packmol software (version 18.169). The MM method with 100 steepest descent and 10,000 conjugate gradient steps was used, and then, the MD protocol consisted of a heating step (100 ps, time step 1 fs, 0 to 300 K), isothermal equilibration (100 ps, 300 K) and production run (5 ns in the NVT ensemble (constant-volume/constant temperature dynamics) at 300 K, coordinates recorded every 10 ps) was employed. Trajectory file data generated from the MD simulation have been used in all the calculations and analyses presented in this research.

Creation of MIPs’ binding sites models included two main stages: prepolymerization complex and polymer chain building and optimization. To obtain the starting structures of prepolymerization mixture models, the boxes with two template molecules (2,4-diamine-1,3,5-triazine) surrounded by eight molecules of appropriate monomer (**1**–**5**) were created. Then, forty molecules of cross-linker, eighteen molecules of acetonitrile and ninety-three molecules of dimethylsulfoxide were added, and complete starting structures were prepared and then optimized according to the MM and MD procedures. The number of reagents and solvent molecules were chosen to mimic their molar ratio, used during the synthetic process.

In the second step, the creation of polymer chains’ models with the binding sites was performed. For that purpose, single bonds between the vinyl groups of the monomer and cross-linker molecules were created in optimized structures of the prepolymerization complexes to form one cross-linked polymeric chain. So-called ESP charges were calculated for the created structures. The MM and MD procedures were repeated for the systems involved the polymeric chain, the template and the solvent to form a specific binding site model in the polymeric matrix.

To analyze the affinity of the polymers towards lamotrigine, adsorption process simulations were performed. Firstly, the template molecules were removed from the models of polymeric cavities, and the empty spaces were proposed as the models of binding sites. Next, the systems consisted of the optimized structures of the polymeric chains, two molecules of the analyte inserted into the models of the MIP cavities (replacing the template), and the solvent, including 418 molecules of methanol and 162 molecules of water, were constructed using Packmol, then the MM and MD simulations were carried out.
